# Thrombomodulin protects against acute vascular and multiorgan injury in sickle cell disease

**DOI:** 10.1172/jci.insight.193884

**Published:** 2025-12-09

**Authors:** Guohui Ren, Dustin R. Fraidenburg, Suman Setty, Jiwang Chen, Janae Gonzales, Maria Armila Ruiz, Zalaya Ivy, Najmeh Eskandari, Richard D. Minshall, James P. Lash, Victor R. Gordeuk, Santosh L. Saraf

**Affiliations:** 1 Division of Hematology and Oncology, Department of Medicine,; 2 Division of Pulmonary, Critical Care, Sleep and Allergy, Department of Medicine,; 3 Department of Pathology,; 4 Cardiovascular Research Core,; 5 Departments of Anesthesiology and Pharmacology, College of Medicine, and; 6 Division of Nephrology, Department of Medicine, University of Illinois Chicago, Chicago, Illinois, USA.

**Keywords:** Hematology, Nephrology, Thrombomodulin

## Abstract

Vaso-occlusive episodes (VOEs) in the setting of hyperhemolysis can rapidly evolve into multiorgan failure in sickle cell disease (SCD). Although the mechanisms for rapid progression to multiorgan failure are unclear, a systemic vasculopathy with thrombotic microangiopathy–type features has been described. Reduced thrombomodulin (TM) function is implicated in some thrombotic microangiopathy syndromes. We observed a greater decline in platelet count and hemoglobin concentration and an increase in vascular injury biomarkers within 24 hours of admission for a VOE in 12 patients with SCD with multiorgan failure versus 12 patients without multiorgan failure. We observed decreased TM expression on the lung and kidney vasculature of 3 additional patients with SCD with multiorgan failure compared with a control patient without SCD. Transgenic SCD mice challenged with cell-free hemoglobin had reduced TM function, increased vascular injury biomarkers, and reduced renal cortical blood flow. Infusion of recombinant TM 2 or 24 hours after the challenge restored cortical blood flow and mitigated increases in vascular injury, complement activation, and tubular injury biomarkers, and protected against acute kidney and lung injury. We demonstrated that impaired TM function may be involved in the systemic vasculopathy of SCD-related multiorgan failure, and infusion of recombinant TM may restore vascular function and protect against acute organ damage.

## Abstract 2

In patients and transgenic sickle mice, thrombomodulin loss is associated with vascular, kidney, and lung injury and rescue with recombinant thrombomodulin mitigates the injury.

## Introduction

Acute vaso-occlusive episodes (VOEs) are a hallmark feature of sickle cell disease (SCD) that can rapidly progress to multiorgan failure. The pathophysiology of this rapid progression is unclear but has been observed in the setting of hyperhemolysis or acute chest syndrome and contributes to approximately 22%–26% of deaths in adults with SCD (1, 2). Several organ systems are affected, and acute kidney injury and acute lung injury are common features of this multiorgan failure syndrome (3).

A thrombotic microangiopathy–type phenotype may be a feature of systemic endothelial injury in SCD-related multiorgan failure. Multiorgan failure and rapidly progressive acute chest syndrome in SCD are heralded by an acute worsening in hemolysis and a drop in platelet counts (4–6). Other features that overlap with the classical pentad of a thrombotic microangiopathy include fever, acute kidney injury, and encephalopathy (3–7). Thus, mechanisms of systemic vasculopathy syndromes, such as atypical hemolytic uremic syndrome, thrombotic thrombocytopenic purpura, and disseminated intravascular coagulation (DIC), may be implicated in the multiorgan failure syndrome of SCD.

Thrombomodulin (TM) is a transmembrane glycoprotein that plays a critical role in maintaining microvascular health. Key functions of TM include sequestering thrombin from the circulation, increasing protein C activation, and regulating complement system activation (8). Missense mutations in the TM gene, *THBD*, diminish the capacity of TM to protect against activated complement and occur in 5% of patients with atypical hemolytic uremic syndrome (9). Reduced TM in the vasculature has also been implicated in thrombotic thrombocytopenic purpura, DIC, preeclampsia, and sepsis, with greater loss of TM being associated with reduced survival in these conditions (10–13). In chimeric mice with a mosaic pattern of TM expression on the vascular endothelium, fibrinogen deposition occurs primarily in TM-deficient regions after hypoxic challenges (14). In mice with targeted mutations in the EGF-like domain of TM, fibrinogen deposition is increased on the vasculature at baseline, and mortality is increased after ischemia-reperfusion challenges compared with wild-type mice (15, 16). Replacement of TM in the setting of systemic vasculopathies may protect against acute organ injury. In a meta-analysis of 2,296 patients with sepsis-related DIC, infusion of intact recombinant TM reduced mortality by 46% and increased the odds of DIC resolution by 2.9-fold without an increase in bleeding events compared with no recombinant TM infusion (17). Treatment of rats with recombinant TM after suprarenal aortic clamping reduced the degree of renal dysfunction, tubular pathology, and mortality compared with untreated rats (18).

Our prior work has demonstrated that cell-free hemoglobin mediates loss of TM and its protective function on endothelial cells in vitro, and that increased fragmented TM is released into the circulation of patients with SCD during multiorgan failure episodes (7). Based on these observations, we investigated whether (a) TM is reduced in the kidney and lung vasculature of patients with SCD with multiorgan failure, (b) cell-free hemoglobin reduces TM function, and (c) infusion of intact recombinant TM after a cell-free hemoglobin challenge can mitigate vascular and acute organ injury in SCD mice.

## Results

### Worsening anemia, thrombocytopenia, and vascular injury biomarkers in patients with SCD and multiorgan failure.

**Figure 1:**
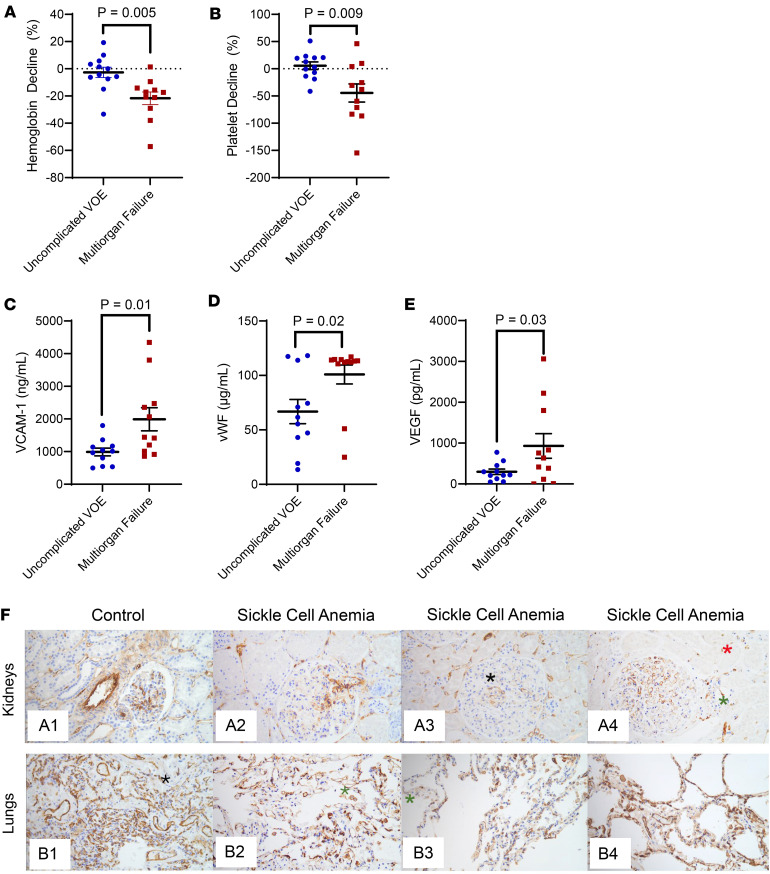
Thrombotic microangiopathy–type features in patients with SCD. Patients with SCD who develop multiorgan failure had a larger (**A**) red blood cell hemoglobin and (**B**) platelet count decline relative to the most recent preceding outpatient laboratory values and higher (**C**) VCAM-1, (**D**) vWF, and (**E**) VEGF compared with those hospitalized for a VOE without multiorgan failure by 1-way ANOVA. (**F**) Kidney section from a resection control (A1) and autopsy specimens of patients with SCD (A2–A4) (original magnification, ×200); stained with TM. The kidneys of patients with SCD show decreased expression (weak to moderate patchy positivity) in the endothelial cells of the glomeruli (black *), arterioles (green *), and peritubular capillaries (red *) compared with the control (A1). Lung section from a resection control (B1) and autopsy specimens of patients with SCD (B2–B4) (original magnification, 200×); stained with TM. The lungs of patients with SCD show decreased expression (weak to moderate patchy positivity) in the endothelial cells in the alveolar spaces (green asterisk) compared with the control (black asterisk) (B1).

This study sample consisted of 12 patients with SCD who developed multiorgan failure and had blood samples available within 24 hours of hospitalization for a VOE between September 2020 and April 2022 (Supplemental Table 1; supplemental material available online with this article; https://doi.org/10.1172/jci.insight.193884DS1). All 12 patients developed acute kidney injury; 7 had acute chest syndrome with respiratory failure, 5 had hepatopathy, and 5 had encephalopathy during the multiorgan failure event. Laboratory features of thrombotic microangiopathy (decline in hemoglobin concentration and platelet count from baseline values) were more significant in patients with SCD who developed multiorgan failure (hemoglobin: –1.4 g/dL [IQR, –1.2 to –1.6 g/dL]; platelet count: –64 × 10^9^ cells/L [IQR, –13 × 10^9^ to –95 × 10^9^ cells/L]) compared with 12 patients matched by age, sex, and SCD genotype admitted for an uncomplicated VOE during the same time period (hemoglobin: –0.2 g/dL [IQR, –0.6 to 0.4 g/dL]; platelet count: 18 × 10^9^ cells/L [IQR, –39 × 10^9^ to 69 × 10^9^ cells/L]) (Figure 1, A and B). Patients with multiorgan failure had similar rates of i.v. volume resuscitation prior to lab collection (multiorgan failure: 538 mL [IQR, 188–1,025 mL]; uncomplicated VOE: 550 mL [IQR, 188–1,650 mL]; *P* = 0.6) and interval of days between the hospitalized versus preceding outpatient lab values (multiorgan failure: 16 [IQR 9–50] days; uncomplicated VOE: 34 [IQR 21–42] days; *P* = 0.5). Vascular injury biomarkers were significantly higher in patients with SCD who developed multiorgan failure compared with patients without multiorgan failure: VCAM-1 was 1,438 ng/mL (IQR, 1,058–2,444 ng/mL) versus 1,009 ng/mL (IQR, 610–1,160 ng/mL), respectively; vWF was 113 μg/mL (IQR, 111–114 μg/mL) versus 62 μg/mL (IQR, 44–104 μg/mL), respectively; and VEGF was 696 pg/mL (IQR, 251–1,900 pg/mL) versus 222 pg/mL (IQR, 148–414 pg/mL), respectively (Figure 1, C–E).

### Reduced TM in kidneys and lungs during multiorgan failure.

We identified 3 additional patients with SCD who had an autopsy between January 2019 and December 2023 during a hospitalization for a VOE complicated by multiorgan failure. All 3 patients had hemoglobin SS genotype, and their hospital courses were complicated by acute kidney injury and acute respiratory failure in the setting of acute chest syndrome. The kidney and lung tissue demonstrated loss of TM on the vascular endothelium, based on a weak to patchy pattern of TM expression, compared with healthy control tissue (Figure 1F). Thrombi in the pulmonary arteries were observed in 2 patients with SCD in regions of reduced endothelial TM.

### TM rescues the vasculature from cell-free hemoglobin-mediated injury in transgenic SCD mice.

**Figure 2:**
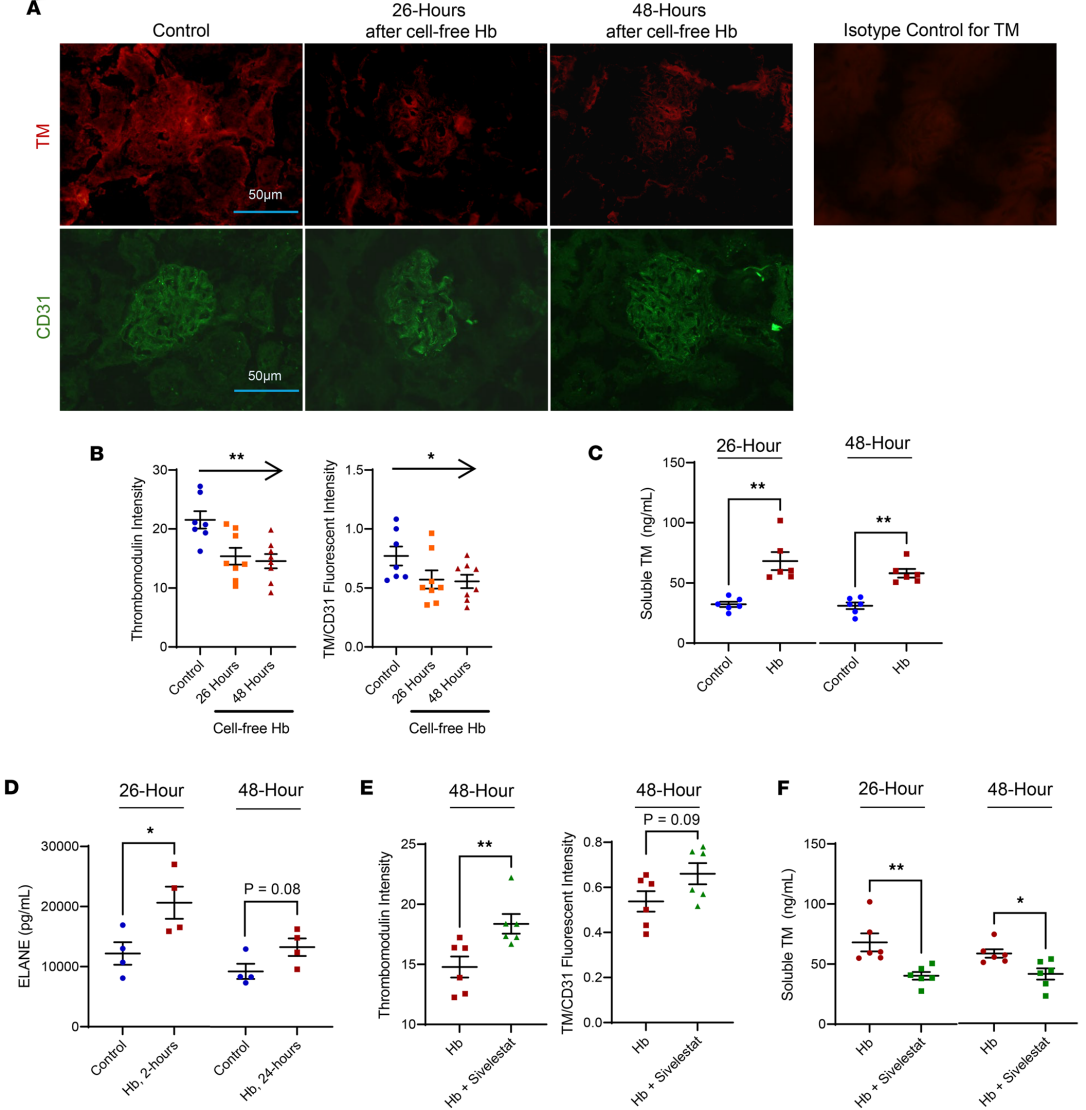
Cell-free hemoglobin reduces TM expression and increases circulating concentrations of soluble TM and neutrophil elastase in SCD mice. (**A** and **B**) TM expression was reduced in the glomerular endothelium relative to CD31 expression (*n* = 8; 4 male, 4 female mice per condition) (original magnification, 600×) by the test for linear trend while (**C**) increased concentrations of soluble TM were released in circulation from control to 26 and 48 hours after cell-free hemoglobin exposure (*n* = 6; 3 male, 3 female mice per condition; Mann-Whitney *U* test). (**D**) Circulating concentrations of neutrophil elastase (ELANE) were increased at 26 hours and trended higher at 48 hours after the cell-free hemoglobin challenge (*n* = 4; 2 male, 2 female mice per condition; 1-way ANOVA). (**E**) Administration of sivelestat, an ELANE inhibitor, led to retained TM on the vasculature by fluorescent intensity at 48 hours after cell-free hemoglobin challenge (1-way ANOVA) and (**F**) reduced soluble TM released into circulation (*n* = 6; 3 male, 3 female mice per condition; Mann-Whitney *U* test). Mice were challenged with a cell-free hemoglobin dose of 0.24 g/kg, and samples and tissue were harvested 26 and 48 hours later. Scale bars: 50 μm. *P < 0.05, **P < 0.01.

Next, we conducted experiments in the transgenic SCD Townes mouse model to investigate the effects of cell-free hemoglobin challenge on TM function and of recombinant TM rescue on biomarkers of vascular, kidney, and lung function. The median plasma concentrations of (a) cell-free hemoglobin (control: 146 mg/L [IQR, 124–172 mg/L]; hemoglobin challenge: 585 mg/L [IQR, 556–652 mg/L]) and (b) heme (control: 57 μM [IQR, 46–63 μM]; hemoglobin challenge: 119 μM [IQR, 110–129 μM]) in the transgenic SCD mice 5 minutes after infusion of i.v. hemoglobin represented 4- and 2-fold increases in cell-free hemoglobin and heme, respectively, similar to the degree of elevations observed in the plasma of patients with SCD at steady-state versus during a VOE or acute chest syndrome event (3, 19–21) (Supplemental Figure 1, A and B). Transgenic SCD mice challenged with cell-free hemoglobin demonstrated reduced TM expression on the glomerular endothelium (Figure 2, A and B) and increased soluble TM released in circulation (Figure 2C). The SCD mice also had increased concentrations of neutrophil elastase, a serine protease released by activated neutrophils that cleaves TM from the endothelium (22), in circulation after exposure to cell-free hemoglobin (Figure 2D). Administration of sivelestat, a neutrophil elastase inhibitor, before and after exposure to the cell-free hemoglobin challenge led to increased TM retained on the glomerular vasculature (Figure 2E and Supplemental Figure 2) and reduced soluble TM released in circulation (Figure 2F).

**Figure 3:**
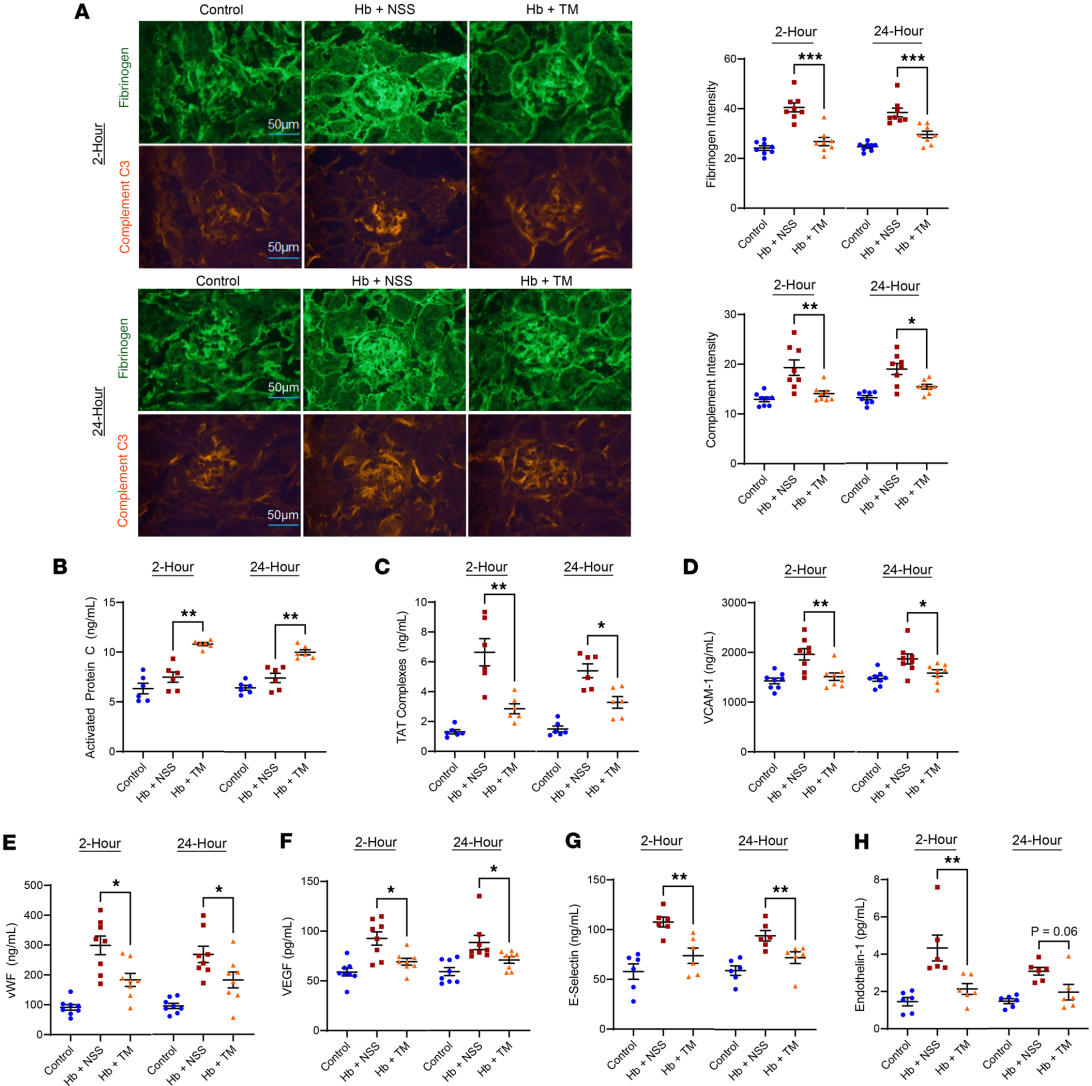
Cell-free hemoglobin reduces TM function, which can be restored with recombinant TM rescue 2 or 24 hours after the cell-free hemoglobin challenge in SCD mice. (**A**) Fluorescence intensity of fibrinogen and C3 complement were increased in cell-free hemoglobin–challenged SCD mice; levels were reduced after rescue with TM, similar to control conditions (*n* = 8; 4 male, 4 female mice per condition; Mann-Whitney *U* test) (original magnification, ×400). (**B**) Activated protein C concentrations were unchanged with cell-free hemoglobin challenge and increased with TM rescue and (**C**) thrombin-antithrombin complexes increased with cell-free hemoglobin challenge and were improved with TM rescue (*n* = 6; 3 male, 3 female per condition; Mann-Whitney *U* test). Increases in vascular injury biomarkers (**D**) VCAM-1, (**E**) vWF, (**F**) VEGF, (**G**) E-selectin, and (**H**) endothelin-1 were observed in cell-free hemoglobin–challenged mice; those that received TM rescue had levels similar to control conditions (**D**–**F**: *n* = 8, 4 male, 4 female mice per condition; **G** and **H**: *n* = 6, 3 male, 3 female per condition; Mann-Whitney *U* test). Mice were challenged with cell-free hemoglobin (0.24 g/kg i.v.) and rescued with TM (5 mg/kg s.c. + 1 mg/kg i.v.) under the respective conditions, and samples and tissue were harvested 24 hours after TM rescue (26 hours for the 2-hour TM rescue and 48 hours for the 24-hour TM rescue after cell-free hemoglobin challenges). Scale bars: 50 μm. Median and IQR values provided in Supplemental Table 2; *P < 0.05, **P < 0.01, ***P < 0.001.

Reduced TM function, as determined by increased fibrinogen and complement C3 intensity in the glomerular vasculature, was observed in the cell-free hemoglobin–challenged mice versus control SCD mice (Figure 3A). Administration of TM 2 or 24 hours after the cell-free hemoglobin challenge mitigated the intensity of fibrinogen and C3 in the glomerular vasculature. Consistent with the antithrombotic effect of TM, we observed increased activated protein C and reduced thrombin-antithrombin complex concentrations in the plasma with TM administration at 2 or 24 hours after the cell-free hemoglobin challenge (Figure 3, B and C). Vascular injury biomarkers VCAM-1, vWF, VEGF, soluble E-selectin, and endothelin-1 were increased in the circulation of cell-free hemoglobin–challenged SCD mice; these biomarkers were reduced after TM administration at 2 or 24 hours after challenge to levels similar to control SCD mice (Figure 3, D–H). We did not observe increased TM expression on the endothelial surface after the TM infusion (Supplemental Figure 3), suggesting that the protective effects against thrombotic, complement, and vascular injury were mediated by the increase in circulating intact recombinant TM.

**Figure 4:**
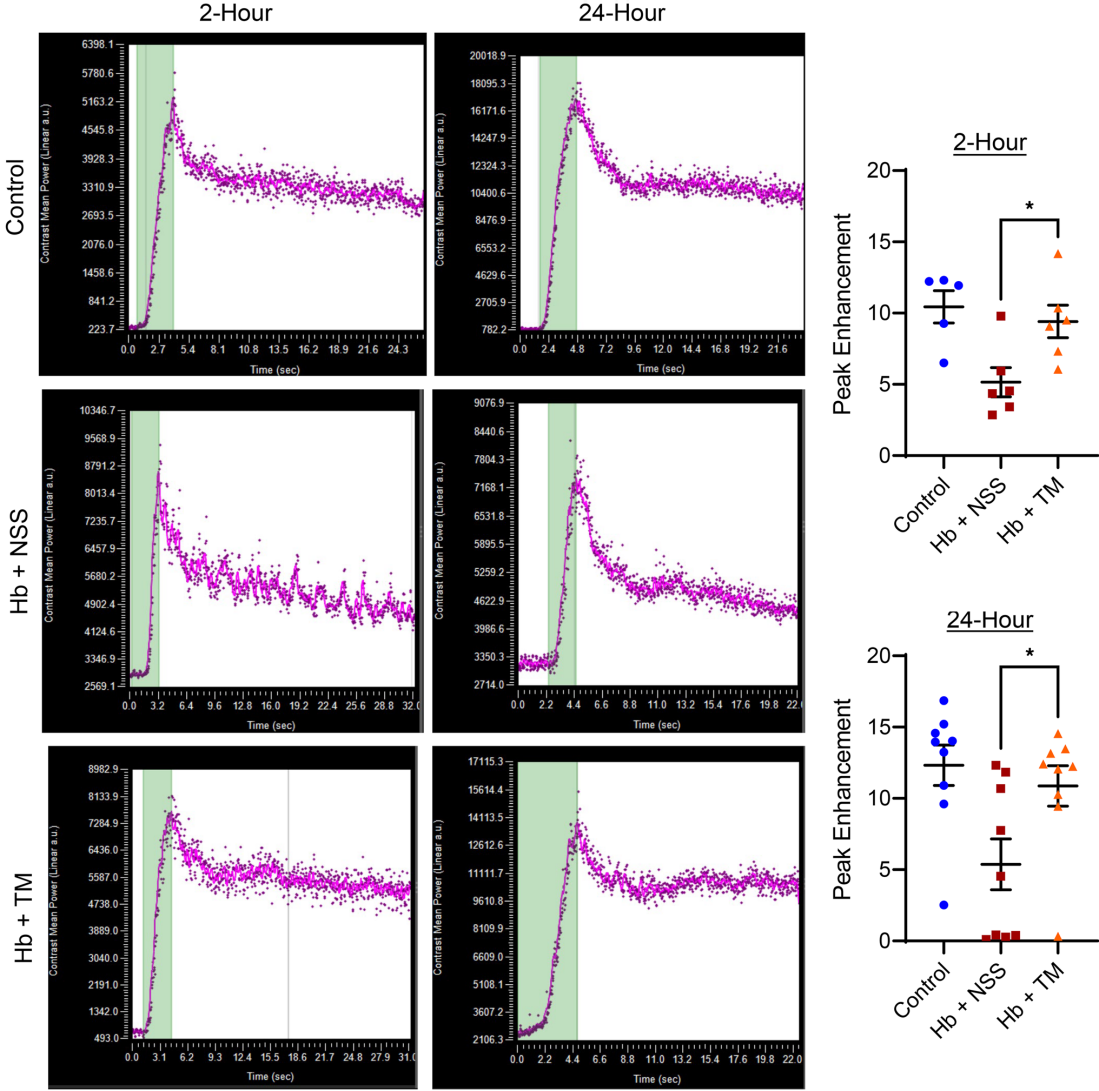
Administration of TM 2 or 24 hours after a cell-free hemoglobin challenge recovers renal cortical perfusion. The images on the left are representative graphs for contrast-enhanced renal cortical perfusion (*n* = 6; 3 male, 3 female mice per condition at 2 hours; *n* = 9; 4 male, 5 female at 24 hours per condition). Peak enhancement, calculated by the ratio of the plateau to baseline value, was reduced 26 or 48 hours after a cell-free hemoglobin challenge compared with SCD mice receiving TM rescue or control conditions (Mann-Whitney *U* test). Mice were challenged with cell-free hemoglobin (0.24 g/kg i.v.) and rescued with TM (5 mg/kg s.c. + 1 mg/kg i.v.) under the respective conditions, and renal cortical perfusion was measured 24 hours after TM rescue (26 hours for the 2-hour TM rescue and 48 hours for the 24-hour TM rescue after cell-free hemoglobin challenge). Median and IQR values provided in Supplemental Table 2; *P < 0.05.

To evaluate the in vivo effects of cell-free hemoglobin and TM rescue on vascular function, we measured cortical blood flow by microbubble contrast–enhanced ultrasound. The peak enhancement, a measure of cortical blood volume that reflects vascular function, was reduced after the cell-free hemoglobin challenge (Figure 4). Administering TM at 2 or 24 hours after challenge led to restoration of cortical blood volume to levels that were similar to control SCD mice.

### TM protects against cell-free hemoglobin-mediated kidney and lung injury in transgenic SCD mice.

**Figure 5:**
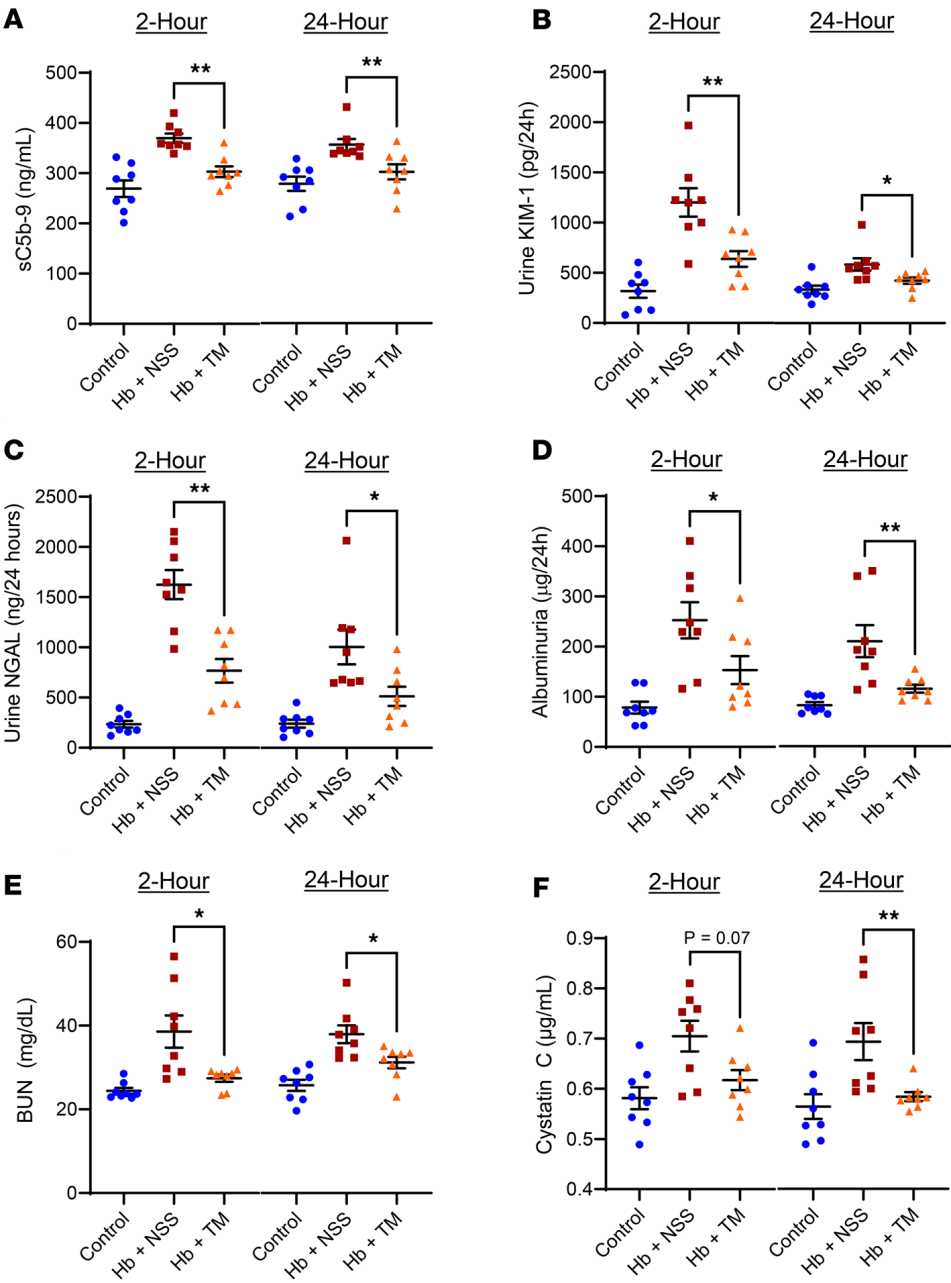
Biomarkers of complement activation, tubular injury, and acute kidney injury were abated with TM rescue at 2 or 24 hours after a cell-free hemoglobin challenge. Biomarkers of (**A**) complement activation (sC5b-9) in circulation and tubular injury (**B**) KIM-1 and (**C**) NGAL in the urine were increased after the cell-free hemoglobin challenge and abated with recombinant TM therapy (*n* = 8; 4 male, 4 female mice per condition; Mann-Whitney *U* test). (**D**–**F**) Kidney function was assessed by urine albumin concentration, serum BUN, and serum cystatin-C, which were increased after the cell-free hemoglobin challenge, consistent with acute kidney injury, whereas TM administered 2 or 24 hours after the challenge was renoprotective (*n* = 8; 4 male, 4 female mice per condition; Mann-Whitney *U* test). Mice were challenged with cell-free hemoglobin (0.24 g/kg i.v.) and rescued with TM (5 mg/kg s.c. + 1 mg/kg i.v.) under the respective conditions, and samples and tissue were harvested 24 hours after the TM rescue (26 hours for the 2-hour TM rescue and 48 hours for the 24-hour TM rescue after cell-free hemoglobin challenges). Median and IQR values provided in Supplemental Table 2; *P < 0.05, **P < 0.01.

Cell-free hemoglobin–challenged SCD mice demonstrated increased complement activation, reflected by sC5b-9 (Figure 5A), as well as increased urine concentrations of tubular injury biomarkers, kidney injury molecule-1 (KIM-1) and neutrophil gelatinase-associated lipocalin (NGAL) (Figure 5, B and C). Treatment of the SCD mice with TM at 2 hours or 24 hours after challenge reduced the levels of sC5b-9, KIM-1, and NGAL to concentrations observed in the non-challenged control SCD mice. The changes in biomarkers of kidney function paralleled the injury biomarkers with acute elevations in urine albumin concentration, serum blood urea nitrogen (BUN), and cystatin C after the cell-free hemoglobin challenge (Figure 5, D–F). These kidney function biomarkers improved after TM rescue and were similar to levels observed in the control conditions.

**Figure 6:**
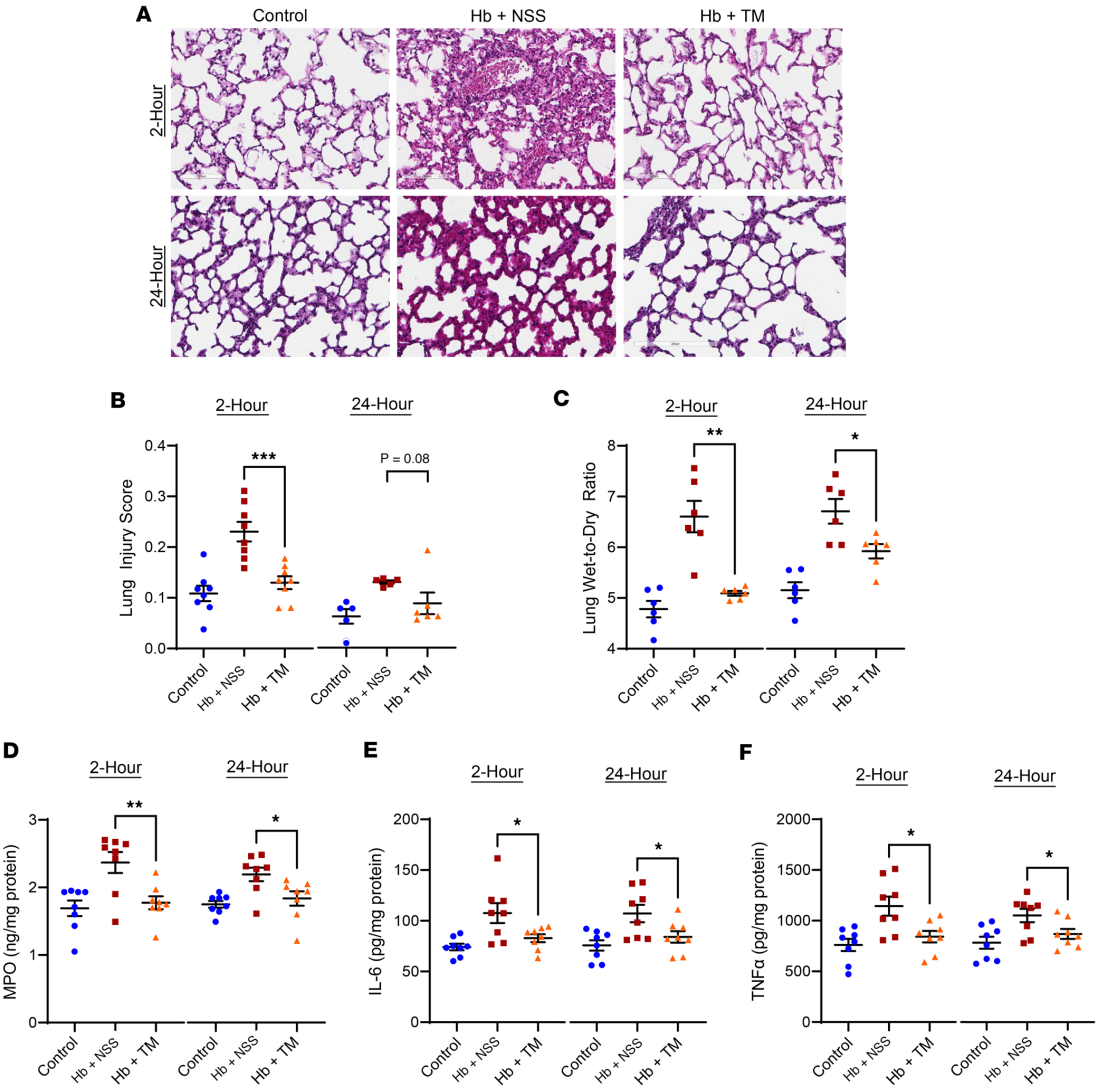
Lung injury improved with TM rescue. (**A** and **B**) Acute lung injury by histopathology elicited after cell-free hemoglobin challenge was improved with TM rescue at 2 or 24 hours after the challenge (*n* = 8, 4 male, 4 female at 2 hours per condition; *n* = 6, 3 male, 3 female at 24 hours per condition; Mann-Whitney *U* test) (original magnification, ×400). (**C**) Lung wet-to-dry ratios increased with cell-free hemoglobin challenges and improved with TM rescue (*n* = 6, 3 male, 3 female per condition at 2 and 24 hours; Mann-Whitney *U* test). Biomarkers of (**D**) neutrophil activity (myeloperoxidase [MPO]) and (**E** and **F**) inflammation (IL-6, TNF-α) increased after cell-free hemoglobin exposure and were improved with TM rescue in lung lysates from the SCD mice (*n* = 8, 4 male, 4 female per condition at 2 and 24 hours; 1-way ANOVA). Mice were challenged with cell-free hemoglobin (0.24 g/kg i.v.) and rescued with TM (5 mg/kg s.c. + 1 mg/kg i.v.) under the respective conditions, and samples and tissue were harvested 24 hours after the TM rescue (26 hours for the 2-hour TM rescue and 48 hours for the 24-hour TM rescue after cell-free hemoglobin challenges). Median and IQR values provided in Supplemental Table 2; *P < 0.05, **P < 0.01, ***P < 0.001.

**Figure 7:**
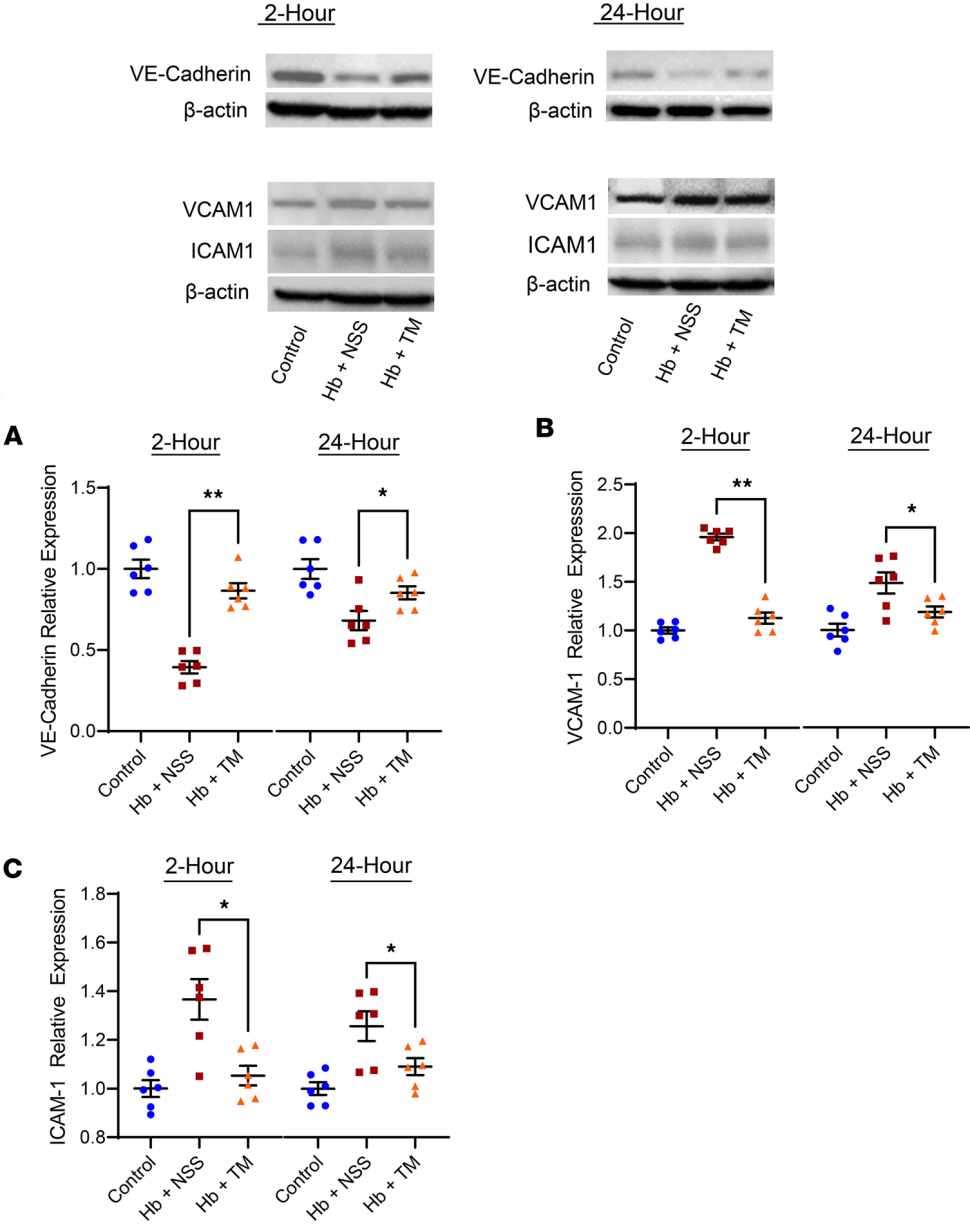
Vascular integrity and injury biomarkers are improved in the lungs of SCD mice treated with TM 2 or 24 hours after a cell-free hemoglobin challenge. The images at the top of the figure are representative Western blots of VE-Cadherin, VCAM-1, and ICAM-1 in the lung lysates under the respective conditions. (**A**) VE-cadherin concentrations were reduced while (**B** and **C**) VCAM-1 and ICAM-1 were increased after cell-free hemoglobin challenges in the lung lysates of SCD mice. VE-cadherin levels improved while VCAM-1 and ICAM-1 were decreased after TM rescue (*n* = 6; 3 male, 3 female mice per condition at 2 and 24 hours; Mann-Whitney *U* test). Mice were challenged with cell-free hemoglobin (0.24 g/kg i.v.) and rescued with TM (5 mg/kg s.c. + 1 mg/kg i.v.) under the respective conditions, and samples and tissue were harvested 24 hours after the TM rescue (26 hours for the 2-hour TM rescue and 48 hours for the 24-hour TM rescue after cell-free hemoglobin challenges). *P < 0.05, **P < 0.01.

Acute lung injury, determined by a composite score of neutrophil infiltration into the alveolar or interstitial space, hyaline membrane formation, proteinaceous debris, and alveolar wall thickening, was evident in the SCD mice challenged with cell-free hemoglobin compared with control SCD mice (Figure 6, A and B). Alveolar barrier dysfunction, as determined by lung wet-to-dry ratio, and biomarkers of neutrophil activity (myeloperoxidase), inflammation (IL-6, TNF-α), and vascular injury (VCAM-1, ICAM-1) were increased, whereas a biomarker of vascular integrity (VE-cadherin) was reduced in the lung lysates of SCD mice after cell-free hemoglobin challenge (Figure 6, C–F, and Figure 7). TM rescue mitigated the degree of lung injury at 2 hours with a trend for improvement at 24 hours after the cell-free hemoglobin challenge (Figure 6, A and B). In parallel, the lung wet-to-dry ratio and biomarkers of injury and vascular integrity improved in the lung lysates of SCD mice that received TM rescue (Figure 6, C–F, and Figure 7).

## Discussion

The median survival of patients with SCD is substantially reduced compared with people without SCD (23). In the Cooperative Study of Sickle Cell Disease, 22% of deaths in adults with SCD occurred during a VOE, with 10% occurring in the setting of acute chest syndrome (1). In a more contemporary cohort of patients with SCD between the ages of 15 and 45 years old, 26% of deaths occurred in the setting of multiorgan failure syndrome, acute chest syndrome, or kidney failure (2). Although there have been advances in treatment to prevent VOE, such as L-glutamine, crizanlizumab, and gene therapy, there is a lack of targeted therapies to prevent the rapid progression of VOE to acute organ failure.

Thrombotic microangiopathy syndromes, such as thrombotic thrombocytopenic purpura, DIC, and atypical hemolytic uremic syndrome, are characterized by systemic vascular damage leading to acute organ injury and are heralded by an acute drop in platelet counts. In a cohort of 173 children and adults with SCD, a decline in platelet count by either 50% or to less than 150 × 10^9^/L predicted a 4.8-fold greater risk of acute chest syndrome rapidly progressing to require 3 L or more of oxygen to maintain oxygen saturations of 90% or higher or mechanical ventilation within 24 hours of symptom onset (4). Rapidly progressing acute chest syndrome was complicated by acute kidney injury in 69%, hepatic dysfunction in 75%, and altered mental status in 44% of patients. In another cohort of 132 patients with SCD, a 20% decline in platelet count within the first 48 hours of admission for VOE along with age, neutrophil count increase, and severe SCD genotype predicted a complicated admission (6). These complicated admissions included 24% with acute chest syndrome and 11% with acute kidney injury. Consistent with a thrombotic microangiopathy, we observed a greater drop in platelet counts in parallel with increased circulating concentrations of vascular injury biomarkers (VCAM-1, vWF, and VEGF) in patients with SCD hospitalized for a VOE with versus without multiorgan failure.

Although systemic thrombotic microangiopathy has been increasingly described in SCD-related acute multiorgan failure, the mechanisms are unclear (24–27). Our data support a role for reduced TM function in the pathophysiology of SCD-related thrombotic microangiopathy. TM is expressed on the endothelium and plays a pivotal role in maintaining microvascular health by negatively regulating thrombin and complement activation. We previously demonstrated that incremental doses of cell-free hemoglobin reduce TM surface expression and TM function in cultured endothelial cells and that hemoglobin SS mice have reduced endothelial-bound TM in the glomerular vasculature compared with hemoglobin AA mice (7, 28). Loss of TM from the vasculature may be mediated by the direct toxic effects of ROS on endothelial cells or by proteolytic cleavage by neutrophil-derived proteases (22). In the current set of experiments, SCD mice challenged with cell-free hemoglobin demonstrated reduced TM expression on the glomerular endothelium along with increased circulating concentrations of neutrophil elastase. Inhibition of neutrophil elastase with sivelestat led to greater retention of TM on the endothelium and less release of soluble TM in circulation. These data suggest that a major mechanism for loss of TM may be mediated through cell-free hemoglobin activation and release of neutrophil elastase. Other potential mechanisms, such as the effects of cell-free hemoglobin and heme on reduced NO signaling and increased ROS formation, may also contribute to the shedding of functional TM from the endothelium and should be explored in future studies. The cell-free hemoglobin–challenged mice also had increased fibrinogen and complement deposition in the glomerular vasculature, consistent with reduced TM function, as well as increased biomarkers of circulating vascular injury (VCAM-1, vWF, VEGF, soluble E-selectin, and endothelin-1) and complement activation (sC5b-9) biomarkers.

Administration of recombinant TM 2 or 24 hours after the cell-free hemoglobin challenge seems to protect the microvasculature, as evidenced by reductions in glomerular vascular fibrinogen and complement deposition as well as reduced biomarkers of vascular injury and complement activation. In vivo vascular function assessed by contrast-enhanced ultrasound demonstrated a reduction in cortical blood flow after cell-free hemoglobin challenge that was restored with recombinant TM infusion. Infusion of TM did not lead to increased TM expression on the vasculature surface, suggesting that infusion of intact recombinant TM in the circulation is providing vascular protective effects. These data suggest that loss of TM function may have an important role in the development of SCD-related microangiopathy.

Recombinant TM is a therapy currently approved to treat sepsis-related DIC in Japan based on a phase III, randomized, double-blind clinical trial demonstrating resolution of DIC in 66.1% of treated patients compared with 49.9% treated with heparin (29). Although another multicenter phase III trial of recombinant human soluble TM failed to demonstrate a statistically significant improvement in 28-day all-cause mortality versus placebo (26.8% vs. 29.4%, respectively), a post hoc analysis demonstrated an 8.3% absolute risk reduction of all-cause mortality in more severe DIC cases, defined by a higher baseline level of coagulation biomarkers or sustained coagulopathy (30). Furthermore, in a meta-analysis of 1,633 patients treated with recombinant TM, patients with sepsis-associated coagulopathy had a 1.3-fold higher 28-day mortality risk compared with patients without sepsis-associated coagulopathy and were the subgroup of patients who demonstrated a survival benefit (RR 0.80, 95% CI: 0.65–0.98) with TM therapy (31). Thrombocytopenia (platelet count < 150 × 10^9^/L) and anemia (Hb < 10.2 g/dL) are reported in approximately 48%–70% and 50% of patients with sepsis at the time of admission to the intensive care unit, respectively, and independently predict 28-day mortality risk (32–38). In our cohort of patients with SCD hospitalized for multiorgan failure, 73% (8 of 11 evaluable) had a decline in platelet count and 91% (9 of 11 evaluable) had a decline in hemoglobin concentration within 24 hours of hospitalization, consistent with a more severe prognosis. The etiology of endothelial injury in sepsis versus SCD-related multiorgan failure may have some similar pathophysiological mechanisms, such as TLR4-mediated activation of NF-Kβ and angiopoietin-2 pathways, as well as nonoverlapping mechanisms, such as pathogen-derived cytokine auto-amplification loops and LPS-mediated endothelial apoptosis, that may lead to differences in recombinant TM response (39, 40). Future studies comparing the loss of vascular TM in patients with SCD-related multiorgan failure to patients without SCD hospitalized for sepsis-associated coagulopathy and sepsis without coagulopathy may help improve our understanding of potential clinical differences in TM response and guide future clinical practice.

We demonstrated that there is patchy loss of TM in the kidneys and lungs of patients with SCD complicated by multiorgan failure, and that recombinant TM protects the kidneys and lungs from acute injury induced by cell-free hemoglobin in SCD mice. The pulmonary vascular beds are susceptible to injury from inflammatory conditions and circulating toxins given that the entire cardiac output circulates to the pulmonary capillaries. Use of recombinant human TM in patients with acute respiratory distress syndrome and DIC substantially improved 60-day survival rates by 47% compared with controls (41). The glomerular vasculature is also sensitive to coagulopathy and complement-mediated damage. In sepsis-induced DIC, recombinant TM reduced the risk of renal replacement therapy dependence at the time of intensive care unit discharge by 57% and was associated with a lower serum creatinine at the time of hospital discharge (42). Our findings support recombinant TM as a potential therapy to ameliorate kidney and lung injury in the setting of SCD-related multiorgan failure and warrant further evaluation in future studies.

Our study is limited by the small sample size from a single center, and the findings will need to be validated in larger multicenter cohorts. Staining for TM on the endothelial surface of kidney and lung tissues was restricted to autopsy samples from patients with VOE and multiorgan failure, and further evaluation in more samples from patients with SCD and a comparison to patients with sepsis without coagulopathy should be evaluated in future studies. Our study focused on the hyperhemolysis model. Investigation of TM function and rescue after challenges that trigger systemic vasculopathy and multiorgan failure, such as hypoxia-reoxygenation or LPS challenge, should be evaluated in future experiments. Damage to the microvasculature is likely multifactorial, including potential contributions of cell-free hemoglobin reducing ADAMTS13 activity and loss of endothelial protein C receptor (43, 44). Whether recombinant TM helps protect against these pathways will also require future investigation.

In conclusion, we found that loss of TM function may be implicated in SCD-related thrombotic microangiopathy and multiorgan failure syndrome. Recombinant TM may have the potential to protect the microvasculature and prevent acute kidney and lung injury for up to 24 hours after hemolytic stress; future studies are warranted to evaluate this therapy in SCD.

## Methods

### Sex as a biological variable.

Sex was considered as a biological variable. We matched patients with SCD with versus without multiorgan failure by sex and performed experiments in the mice using similar numbers of male and female SCD mice.

### Patients with SCD.

We prospectively collected bio-samples from patients with SCD within 24 hours of hospitalization for an uncomplicated VOE or a VOE complicated by multiorgan failure between September 2020 and April 2022. Multiorgan failure was defined as having at least 2 organ systems demonstrating evidence of organ injury, as previously defined (3). Hemoglobin and platelet counts within 24 hours of admission were compared with the most recent outpatient values within 6 months preceding the hospitalization. One patient in the multiorgan failure group did not have baseline labs at our institution to determine the hemoglobin and platelet percentage decline from baseline. We measured the vascular injury biomarkers VCAM-1 (DVC00, Novus Biologicals), vWF (EHVWF, Thermo Fisher Scientific), and VEGF (KHG0111, Thermo Fisher Scientific) by ELISA in all 12 patients with SCD who consented to bio-samples with multiorgan failure syndrome and in patients matched by age, sex, and SCD genotype who were hospitalized with an uncomplicated VOE.

Autopsies performed in 3 patients with SCD during a multiorgan failure event between January 2019 and December 2023 were identified by chart review. Kidney and lung tissues were stained for TM (43514S, Cell Signaling Technology), phosphotungstic acid (AHPPTA125, StatLab) and H&E, and the histopathology was compared with kidney and lung tissue from a non-SCD control. Staining was done on Leica BOND Rx with BOND polymer refine detection kit (DS9800) in the University of Illinois at Chicago (UIC) Research Histology Core. Tissue sections were subjected to heat-based antigen retrieval with BOND Epitope retrieval buffer 1 (pH 6.0, Leica Biosystems, AR9961) for 20 minutes at 99°C and incubated with TM antibody (1:2,000, Cell Signaling Technology, 43514) for 30 minutes at room temperature.

### Transgenic SCD mice.

Transgenic SCD mice (stock 013071, Townes model, The Jackson Laboratory) expressing human SCD hemoglobin with pathophysiological features of SCD (45) were bred and housed in the UIC Biologic Resources Laboratory. This transgenic SCD mouse model was chosen based on prior studies demonstrating that a single hemin infusion leads to an acute lung injury that mirrors clinical features of acute chest syndrome in patients with SCD, including hypoxemia, vascular congestion, edema, and extensive lung damage (46). Studies were conducted in age- and sex-matched hemoglobin SS mice. SCD mice were challenged with cell-free hemoglobin (H7379, Sigma-Aldrich, 0.24g/kg i.v.), a dose shown to induce endothelial dysfunction in non-SCD mice (47), or normal saline solution. This was followed 2 or 24 hours later by either infusion of intact soluble TM (5 mg/kg s.c. and 1 mg/kg i.v., Asahi Kasei Pharma), a dosing schema that was used to protect rats from ischemic kidney injury (18), or normal saline. These time points were chosen to evaluate the effects of recombinant TM acutely (2 hours) and to provide a therapeutic window for when patients may present for emergent care or when daily clinical laboratory testing may detect acute kidney injury in hospitalized patients (24 hours). For the neutrophil elastase inhibition experiments, sivelestat (HY-17443, MedChemExpress LLC), a specific neutrophil elastase inhibitor, was utilized. Mice were treated with either sivelestat (100 mg/kg) or vehicle i.p. 12 hours and 0.5 hours before hemoglobin injection and every 12 hours thereafter.

Urine samples were collected for 24 hours using metabolic cages. Urine and blood were collected 24 hours after the infusion of intact soluble TM or normal saline. Blood collection was performed via retro-orbital bleeding into K2EDTA tubes (365974, Becton, Dickinson and Company) under isoflurane anesthesia. The mice were euthanized immediately after blood collection for histopathological evaluation.

ELISA kits were used according to the manufacturer’s instructions to measure mouse albumin (E99-134, Bethyl Laboratories), KIM-1 (ab119596-KIM-1 [TIM-1] Mouse ELISA kit, Abcam), and NGAL (ab199083, Abcam) urine concentrations. Plasma concentrations of hemoglobin (E88-134, Bethyl Laboratories), TM (ab209880, Abcam), neutrophil elastase (MELA20, R&D Systems), activated protein C (ABIN6953414, Antibodies-online Inc.), thrombin-antithrombin complexes (TAT, ab230933, Abcam), VCAM-1 (ab100750, Abcam), vWF (NBP2-68175, Novus Biologicals), VEGF (MMV00, R&D Systems), E-selectin (DY575, R&D Systems), endothelin-1 (DET100, R&D Systems), soluble C5b-9 complex (E-EL-M1129, Elabscience), and cystatin C (EMCST3, Invitrogen) were measured by ELISA. Plasma concentrations of heme were measured with a heme assay kit (MAK316, Sigma-Aldrich). Urea concentration was measured using a BUN detection kit (EIABUN, BUN Colorimetric Detection kit, Invitrogen).

Renal tissue was frozen in OCT compound and cryo-sectioned at 4 μm. Sections were fixed with acetone, blocked in 5% BSA, and incubated overnight with primary antibodies at 4°C. Fluorophore-conjugated secondary antibodies were applied, and tissue sections were mounted with antifade mounting medium (ProLong Diamond Antifade Mountant with DAPI, Thermo Fisher Scientific). All washes were done with PBS. Images were taken using an Olympus BX51/IX70 microscope. Quantification of fluorescence was conducted using mean grayscale intensity in Image J (NIH). Ten random glomeruli per mouse kidney section sample were quantified and results averaged for each sample. The primary antibodies were as follows: anti-C3 antibody (PA1-29715, Thermo Fisher Scientific), anti-fibrinogen antibody (ab34269), anti-CD31 antibody (ab281583) (Abcam), and anti-TM antibody (PA5-47550, Thermo Fisher Scientific). For the TM staining isotype control, the primary antibody was goat IgG isotype (02-6202, Thermo Fisher Scientific).

In a separate set of similar experiments, SCD mice received i.v. microbubbles (Vevo MicroMarker contrast) 24 hours after either the TM rescue or normal saline infusion to assess renal cortical blood flow by contrast-enhanced ultrasound (Vevo2100 system). Experiments were performed in the UIC Cardiovascular Core Imaging Center, and peak enhancement, representing the ratio of the plateau to baseline contrast value, was determined using Vevo Lab analysis software to measure perfusion to the kidney cortex.

Acute lung injury was determined by histopathological evidence of tissue injury using the previously defined American Thoracic Society Lung Injury Scoring System (48). Lung tissues were fixed and stained with H&E in a standard fashion. Twenty random high-powered fields were analyzed and independently scored for each sample. A composite score identifying neutrophils in the alveolar space, neutrophils in the interstitial space, deposition of hyaline membrane, proteinaceous debris filling airspaces, and alveolar septal thickening was determined for each field, and the score was averaged over all fields for each individual sample. For lung wet-to-dry weight ratio, the right lung was weighed (wet weight) and then dried at 60°C in an oven for 6 days to obtain the constant weight (dry weight) for the calculation.

Lung tissues were homogenized with a tissue homogenizer (Thermo Fisher Scientific Sonic Dismembrator, model 100) in 5 volumes of lysis buffer containing 0.5% Triton X-100, 150 mM NaCl, 15 mM Tris, 1 mM CaCl, and 1 mM MgCl_2_, pH 7.4 and Halt Protease Inhibitor Cocktail (87786, Thermo Fisher Scientific). Homogenates were then centrifuged at 10,000*g* for 10 minutes. Supernatants were collected, and total protein concentration was measured using the BCA method (23225, Thermo Fisher Scientific). Myeloperoxidase (EMMPO), IL-6 (KMC0061), and TNF-α (501125453, Thermo Fisher Scientific) in the supernatants were measured by ELISA. Protein concentrations of anti–VE-cadherin (ab33168), VCAM-1 (ab134047), and ICAM-1 (ab222736) (all Abcam) were determined by Western blot analysis using β-actin (20536-1-AP, Proteintech) as the reference protein for relative expression.

### Statistics.

Variables were compared by treatment status with the Mann-Whitney *U* test or 1-way ANOVA. The changes in TM and TM-to-CD31 expression under control conditions and at 2 and 24 hours were compared using the test for linear trend. Analyses were performed using Systat 13 (Systat Software Corporation) and GraphPad Prism 9.3. Median values and IQR are provided. *P* < 0.05 was used to determine statistical significance.

### Study approval.

The study was approved by the IRB at UIC prior to obtaining autopsy samples, and participants provided written informed consent in accordance with the Declaration of Helsinki prior to collection of bio-samples. All animal procedures were conducted under protocols approved by the Illinois IACUC at UIC.

### Data availability.

The data that support the findings of this study are available in the Supporting Data Values file.

## Author contributions

GR, DRF, SS, JC, JG, RDM, JPL, VRG, and SLS contributed to study conception and design. GR, DRF, SS, JC, JG, MAR, ZI, NE, RDM, JPL, VRG, and SLS contributed to data collection, analysis, and interpretation of results. GR, DRF, SS, JC, JG, MAR, ZI, NE, RDM, JPL, VRG, and SLS were responsible for draft manuscript preparation. All authors critically reviewed and approved the final version of this manuscript.

## Funding support

This work is the result of NIH funding, in whole or in part, and is subject to the NIH Public Access Policy. Through acceptance of this federal funding, the NIH has been given a right to make the work publicly available in PubMed Central.

- The project described was supported by the NIH through grants R01 HL-153161 and K24 HL-177273 (to SLS), R56 HL-167875 (to DRF), and T32 HL-144909 (to JG).

## Supplementary Material

Supplemental data

Unedited blot and gel images

Supporting data values

## Version 1 12/09/2025

In-Press Preview

## Version 2 01/23/2026

Electronic publication
