# Supplementary material for: Thrombomodulin protects against acute vascular and multiorgan injury in sickle cell disease
Source: JCI Insight. 2025 Dec 9;11(2):e193884. doi: 10.1172/jci.insight.193884 (PMC12892889; doi:10.1172/jci.insight.193884)
Supplement: Supplemental data [file jciinsight-11-193884-s167.pdf]

**Supplementary Table 1:** Characteristics of patients with sickle cell disease who developed multiorgan failure						
**#**	**Age**	**Sex**	**Hemoglobin Genotype**	**SCD Therapy**	**Hydration Pre-labs**	**Complication**
1	25	M	SS	Hydroxyurea	1,000 mL*	AKI stage 3; Hepatopathy
2	45	F	SS	Hydroxyurea	700 mL	AKI stage 1; Acute chest syndrome with worsening oxygen requirement
3	32	M	SS	None	125 mL	AKI stage 2; Hypoxic respiratory failure; Encephalopathy
4	58	F	SS	None	100 mL	AKI stage 1; Encephalopathy
5	53	F	SS	Chronic RBC Exchange	300 mL	AKI stage 1; Hypoxic respiratory failure; Vasopressor support
6	66	F	SS	None	1,000 mL*	AKI stage 2; Encephalopathy
7	49	M	SS	Chronic RBC Exchange	0 mL	AKI stage 1; Acute chest syndrome with intrahepaic cholestasis
8	27	F	SS	None	375 mL	AKI stage 2; Acute chest syndrome with worsening oxygen requirement, intrahepatic cholestasis
9	37	F	SS	Hydroxyurea	250 mL	AKI stage 2; Hepatopathy
10	32	M	SS	Hydroxyurea	1,050 mL	AKI stage 1; Acute chest syndrome with worsening oxygen requirement and non-invasive mechanical ventilation support
11	47	M	SC	None	1,500 mL	AKI stage 2; Hypoxic respiratory failure; Intrahepatic cholestasis
12	32	M	SS	Hydroxyurea	1,200 mL	AKI stage 2; Encephalopathy with seizure

* Hydration given as a bolus

SCD, sickle cell disease; M, male; F, female; AKI, acute kidney injury; RBC, red blood cell

Supplementary Table 2: Data values from the 2- and 24-Hour SCD mice challenged.

	2-Hour			24-Hour		
	**Control**	**Hb + NSS**	**Hb + TM**	**Control**	**Hb + NSS**	**Hb + TM**
**Activated Protein C (ng/mL)**	6.1 (5.1 – 7.4)	7.6 (6.1 – 8.2)	10.9 (10.6 – 11.0)	6.4 (6.0 – 6.8)	7.7 (6.2 – 8.5)	9.8 (9.5 – 10.6)
**TAT complexes (ng/mL)**	1.2 (1.1 – 1.4)	6.3 (5.3 – 9.0)	2.7 (2.4 – 3.4)	1.3 (1.2 – 1.8)	5.6 (4.2 – 6.3)	3.3 (2.2 – 4.3)
**VCAM-1 (ng/mL)**	1459 (1296 – 1524)	2015 (1684 – 2168)	1497 (1343 – 1587)	1484 (1377 – 1542)	1853 (1736 – 1955)	1642 (1460 – 1732)
**vWF (ng/mL)**	90 (75 – 101)	317 (218 – 368)	180 (146 – 226)	90 (79 – 117)	251 (219 – 317)	190 (136 – 219)
**VEGF (pg/mL)**	57 (56 – 64)	97 (75 – 108)	69 (65 – 74)	56 (49 – 71)	80 (77 – 91)	75 (61 – 78)
**E-selectin (ng/mL)**	65 (42 – 72)	108 (103 – 112)	74 (55 – 91)	60 (53 – 69)	92 (86 – 104)	77 (74 – 81)
**Endothelin-1 (pg/mL)**	1.6 (0.8 – 1.9)	3.5 (3.3 – 4.8)	2.0 (1.8 – 2.9)	1.6 (1.2 – 1.7)	3.2 (2.6 – 3.3)	1.6 (1.3 – 2.2)
**Peak Enhancement (dB)**	11.9 (8.6 – 12.2)	4.4 (3.4 – 5.9)	9.3 (7.3 – 10.4)	14.0 (10.6 – 14.7)	4.5 (0.4 – 11.0)	12.2 (10.1 – 13.2)
**sC5b-9 (ng/mL)**	268 (234 – 308)	358 (355 – 387)	298 (286 – 316)	291 (253 – 309)	347 (343 – 363)	309 (280 – 335)
**Urine KIM-1 (pg/24 h)**	354 (130 – 441)	1211 (980 – 1336)	666 (429 – 810)	314 (275 – 372)	559 (478 – 596)	441 (388 – 483)
**Urine NGAL (ng/24 h)**	201 (167 – 310)	1609 (1344 – 1976)	756 (439 – 1101)	237 (157 – 293)	816 (659 – 1188)	458 (282 – 718)
**Albuminuria (µg/24 h)**	80 (55 – 103)	239 (179 – 329)	114 (94 – 215)	76 (67 – 101)	191 (142 – 275)	110 (96 – 131)
**BUN (mg/dL)**	24 (23 – 25)	36 (29 – 47)	28 (26 – 29)	27 (23 – 28)	37 (33 – 40)	33 (30 – 34)
**Cystatin C (µg/mL)**	0.58 (0.54 – 0.62)	0.74 (0.62 – 0.77)	0.61 (0.58 – 0.65)	0.54 (0.51 – 0.61)	0.67 (0.61 – 0.77)	0.58 (0.57 – 0.59)
**Lung Injury Score**	0.11 (0.09 – 0.13)	0.23 (0.18 – 0.29)	0.14 (0.08 – 0.16)	0.08 (0.05 – 0.09)	0.14 (0.13 – 0.14)	0.07 (0.07 – 0.09)
**Lung Wet-to-Dry Ratio**	4.8 (4.5 – 5.2)	6.5 (6.3 – 7.3)	5.1 (5.0 – 5.2)	4.4 (4.2 – 4.9)	6.5 (5.6 – 6.9)	5.6 (5.2 – 5.6)
**MPO (ng/mg protein)**	1.82 (1.52 – 1.93)	2.56 (2.17 – 2.66)	1.77 (1.70 – 1.89)	1.78 (1.66 – 1.85)	2.28 (2.06 – 2.39)	1.94 (1.71 – 2.03)
**IL-6 (pg/mg protein)**	74 (69 – 80)	108 (83 – 120)	86 (75 – 91)	80 (62 – 88)	108 (83 – 129)	83 (73 – 94)
**TNFα (pg/mg protein)**	812 (636 – 885)	1129 (927 – 1360)	855 (733 – 959)	797 (603 – 920)	1127 (867 – 1151)	845 (753 – 955)

Median (interquartile ranges) provided

SCD, sickle cell disease; Hb, hemoglobin; NSS, normal saline solution; TM, thrombomodulin; VCAM-1, vascular cell adhesion molecule-1; vWF, von Willebrand Factor; VEGF, vascular endothelial growth factor; sC5b-9, soluble C5b-9; KIM-1, kidney injury molecule-1; NGAL, neutrophil gelatinase-associated lipocalin; BUN, blood urea nitrogen; MPO, myeloperoxidase; IL-6, interleukin-6; TNFα, tumor necrosis factor α

Supplemental Figure 1: A) Cell-free hemoglobin(Hb) (control: 146 mg/L, interquartile range 124 – 172 mg/L; Hb: 585 mg/L, interquartile range 556 - 652 mg/L) and B) heme concentrations (control: 57 μM, interquartile range 46 – 63 μM; Hb: 119 μM, interquartile range 110 – 129 μM) in the plasma of transgenic sickle mice 5 minutes after infusion of intravenous cell-free hemoglobin (0.24g/kg). (n=12; 6 male, 6 female mice; Mann-Whitney test).

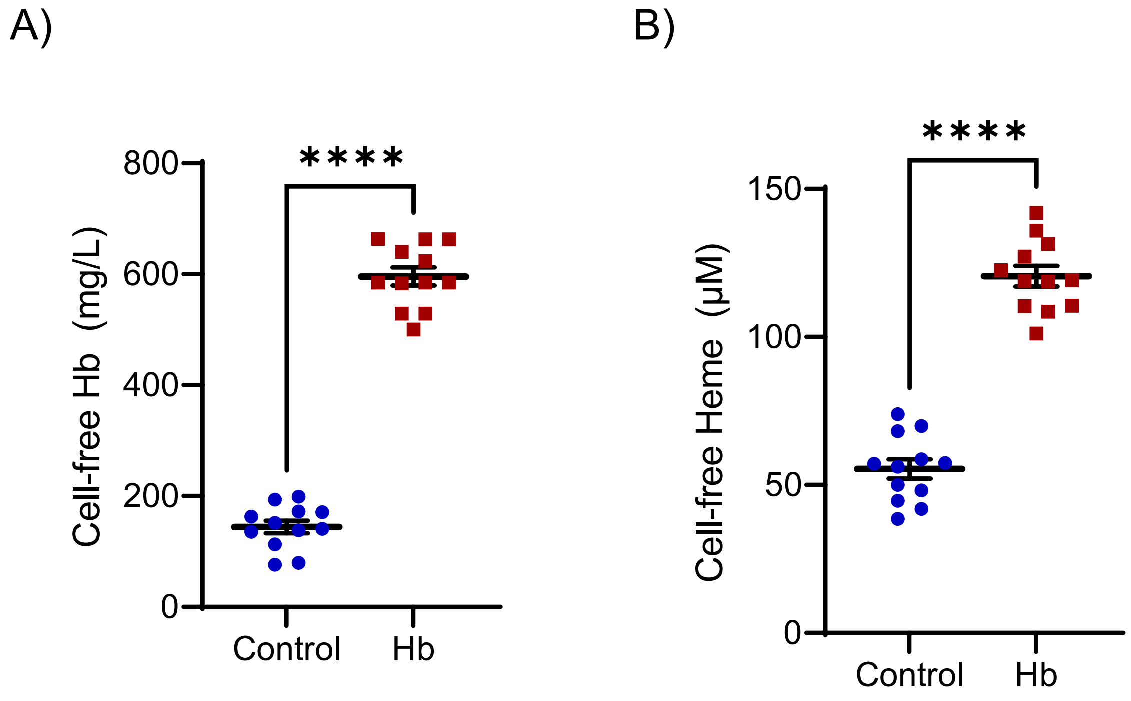

Supplemental Figure 2: Expression of thrombomodulin (TM) and CD31 in the glomeruli of transgenic sickle mice treated with or without sivelestat treatment (100 mg/kg intraperitoneally) 12 and 0.5 hours before and every 12 hours after a cell-free hemoglobin challenge (0.24 mg/kg intravenous). Kidney tissue was harvested 48 hours after the cell-free hemoglobin challenge (n=6; 3 male, 3 female mice). Scale bars represent 50μm.

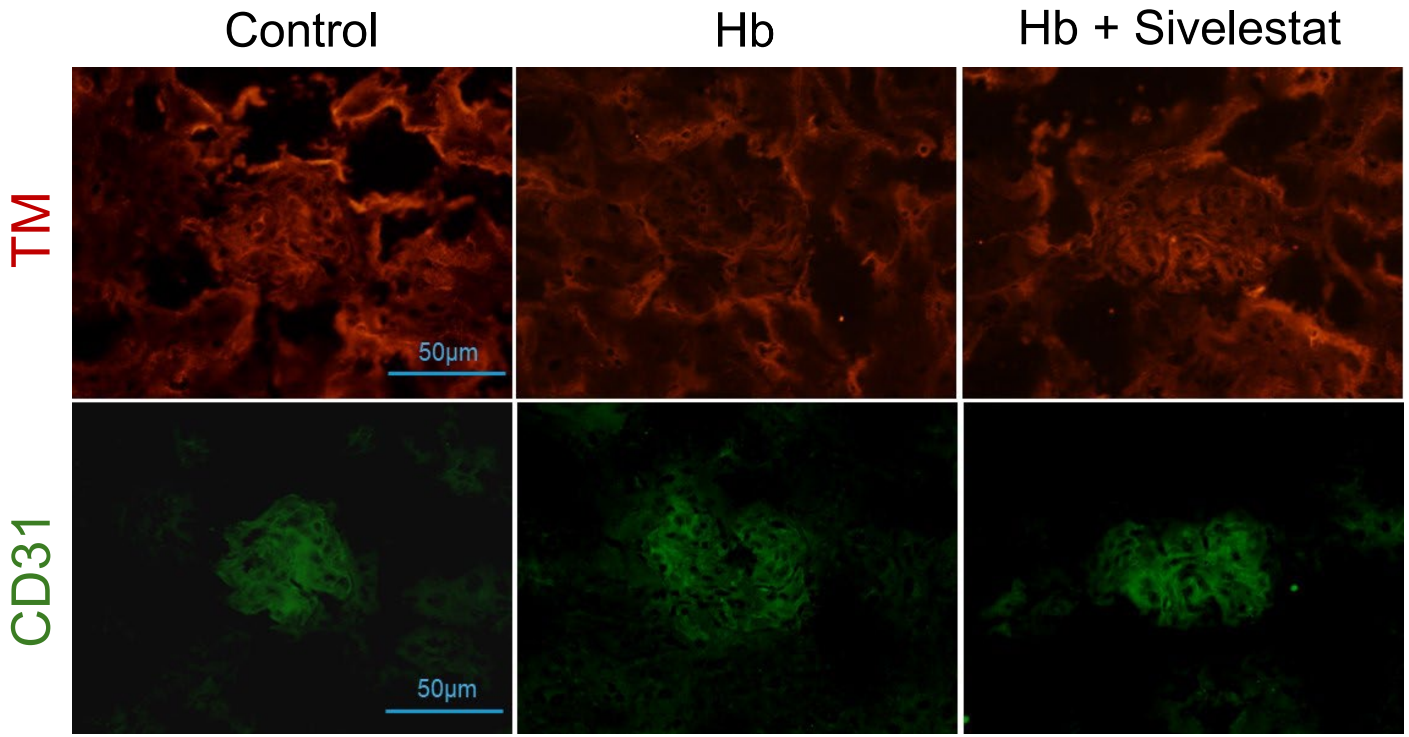

Scale bars represent 50μm.

Supplemental Figure 3: Expression of thrombomodulin (TM) and CD31 in the glomeruli after a cell-free hemoglobin challenge (0.24 mg/kg intravenous) with TM rescue (5 mg/kg subcutaneous + 1mg/kg intravenous) 2- and 24-hours after the cell-free hemoglobin challenge. Kidney tissue was harvested 24 hours after TM rescue (26-hours for 2-hour TM rescue and 48-hours for the 24-hour TM rescue). NSS, normal saline solution (n=8; 4 male, 4 female mice). Scale bars represent 50μm.

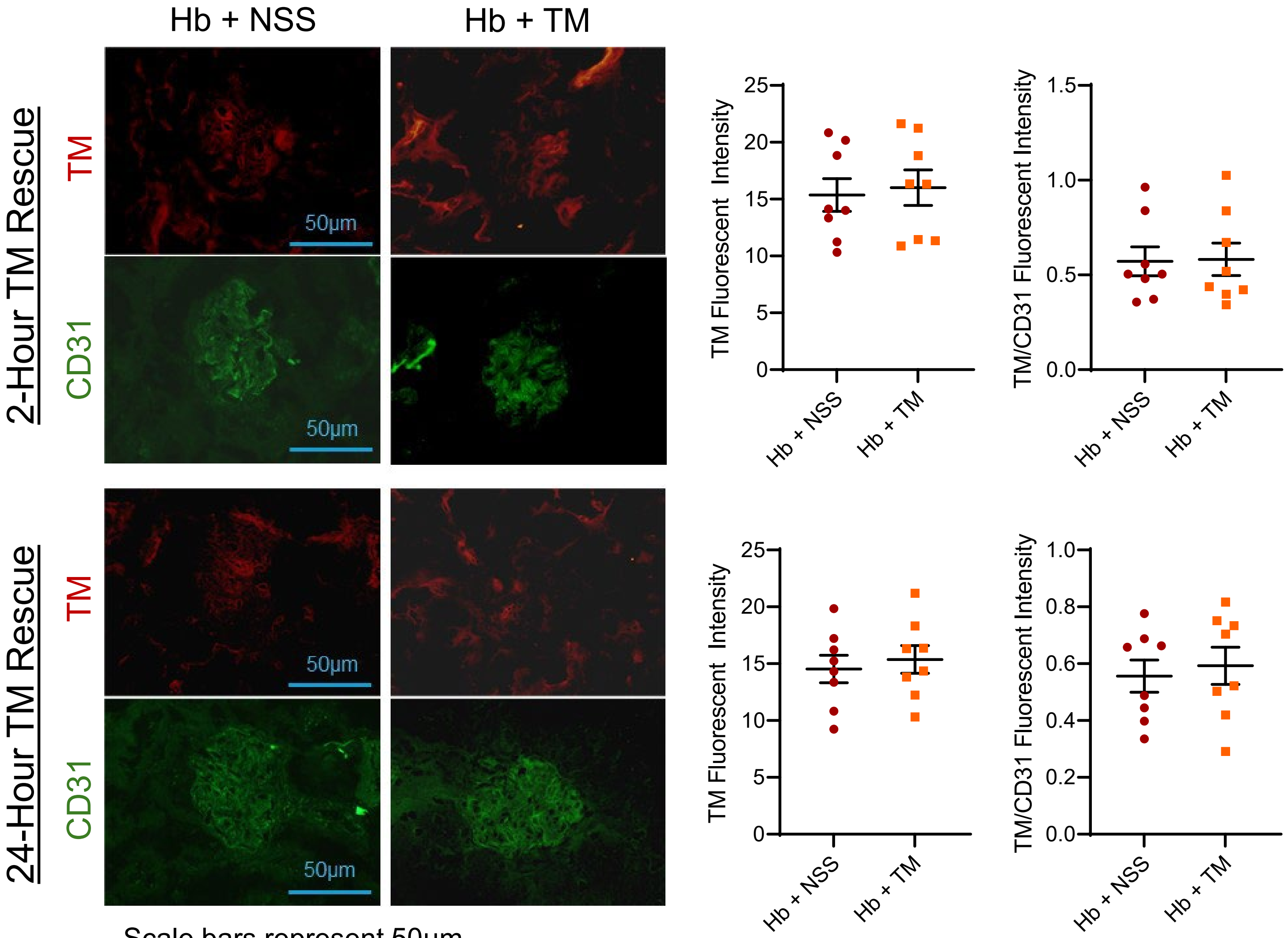

Scale bars represent 50μm.
