# Supplementary material for: Thrombomodulin protects against acute vascular and multiorgan injury in sickle cell disease
Source: JCI Insight. 2025 Dec 9;11(2):e193884. doi: 10.1172/jci.insight.193884 (PMC12892889; doi:10.1172/jci.insight.193884)

Full unedited blots
(2-Hour)

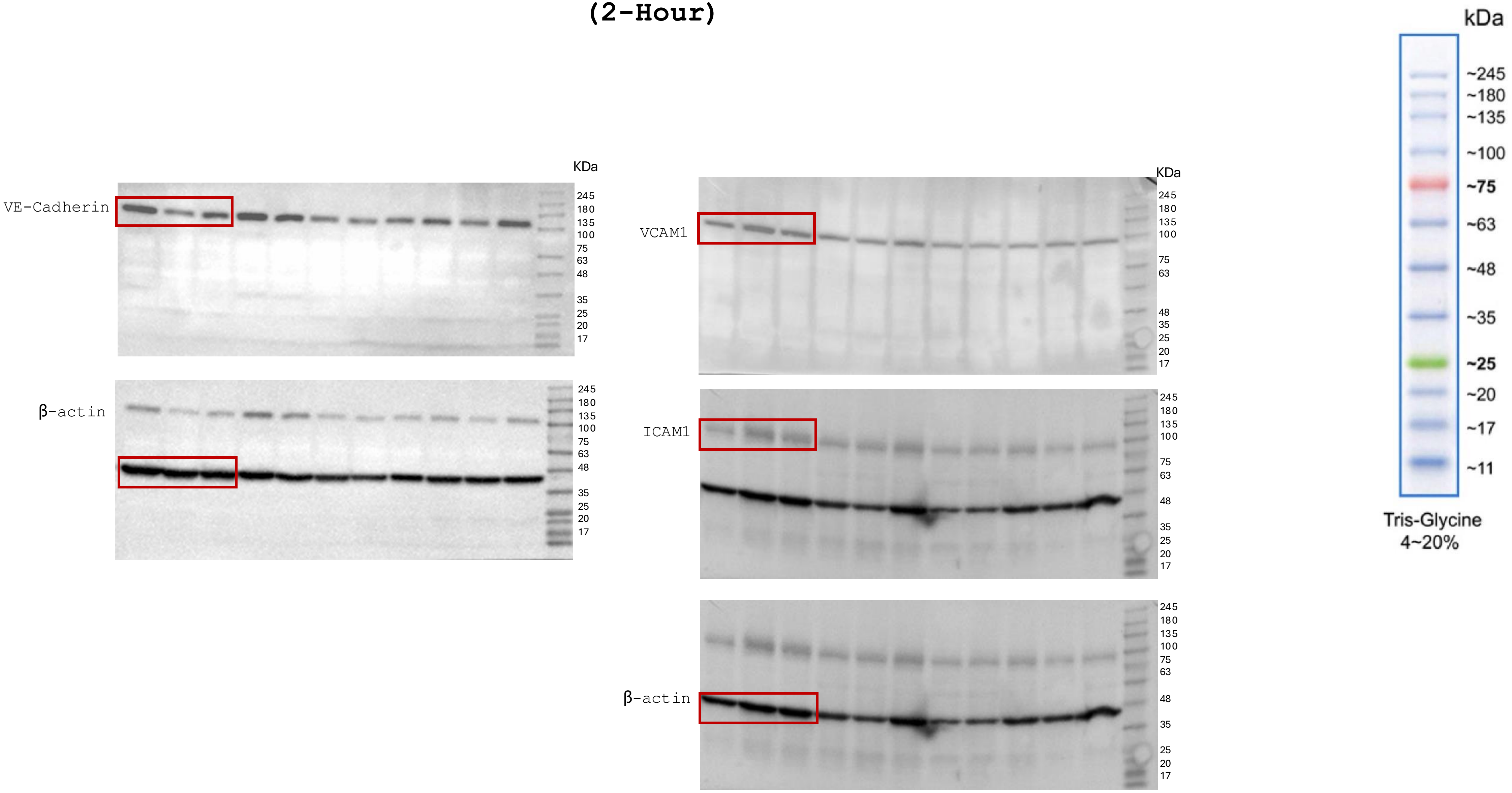

Full unedited blots
(24-Hour)

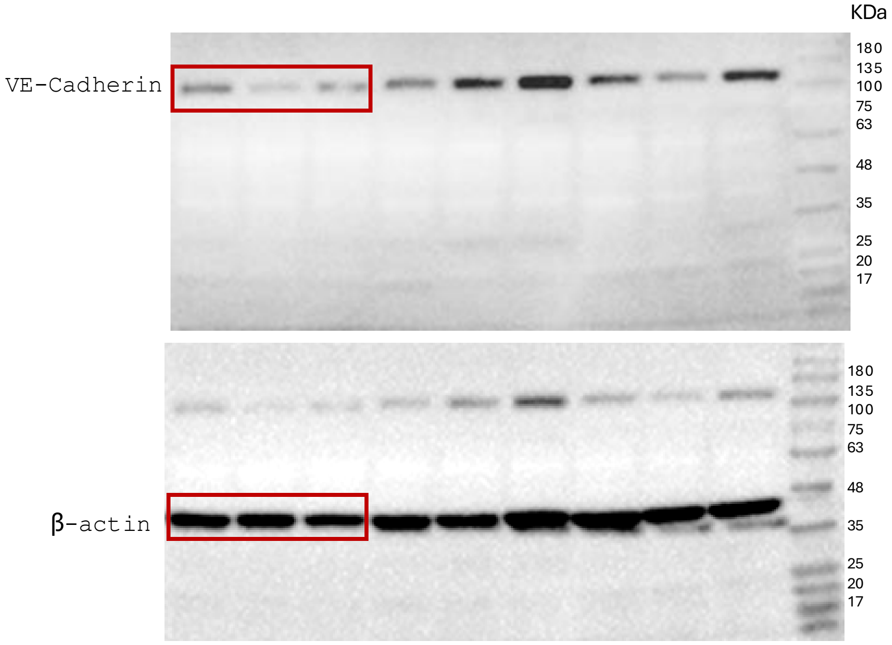

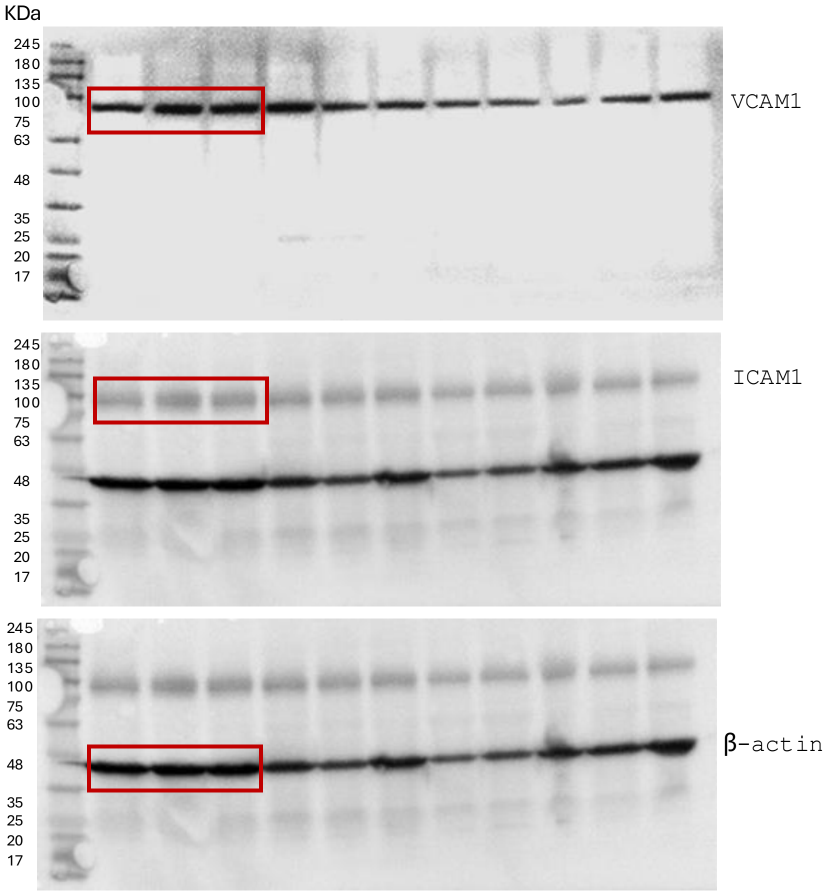

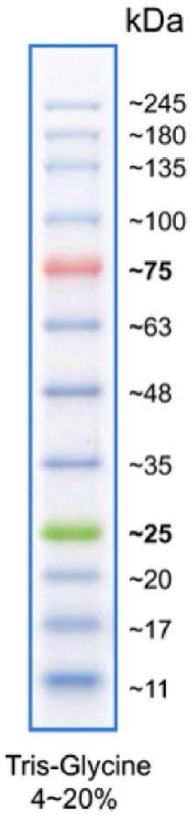

Full unedited blots with all detailed labels

(2-Hour)

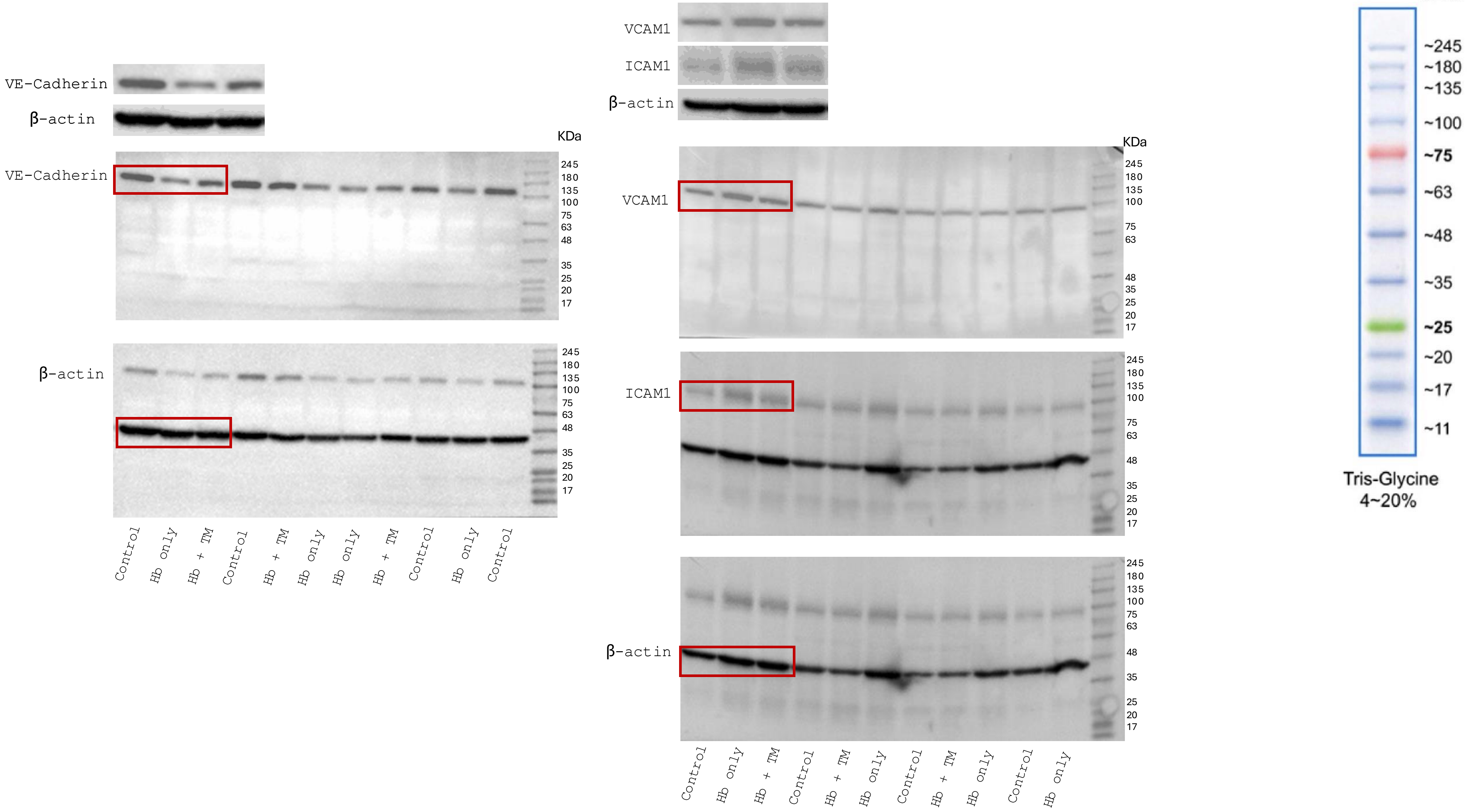

Full unedited blots with all detailed labels

(24-Hour)

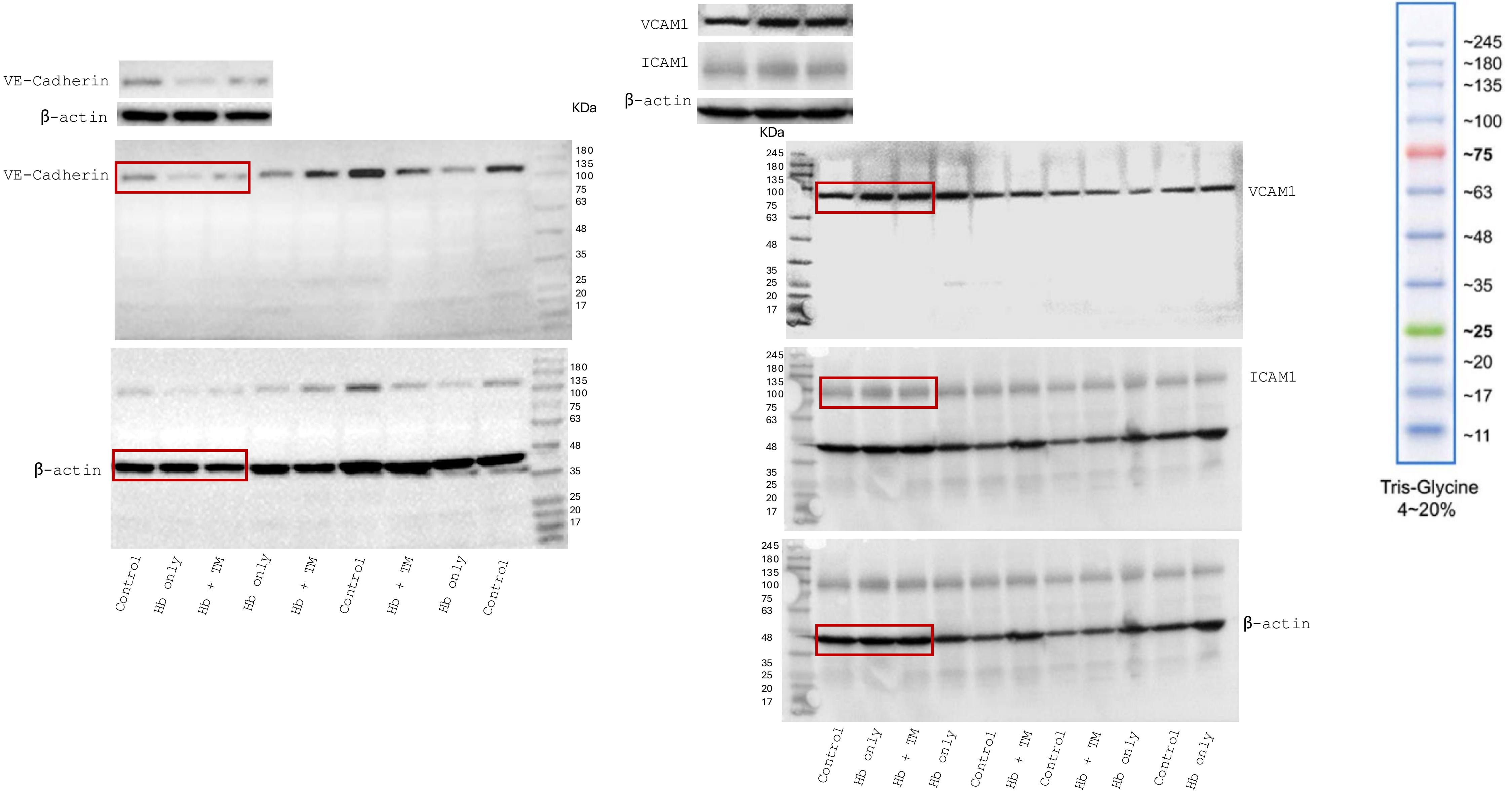

Supplement: Unedited blot and gel images [file jciinsight-11-193884-s168.pdf]
